# Subjective health status and health-related quality of life among women with Recurrent Vulvovaginal Candidosis (RVVC) in Europe and the USA

**DOI:** 10.1186/1477-7525-11-169

**Published:** 2013-10-11

**Authors:** Samuel Aballéa, Florent Guelfucci, Julian Wagner, Amine Khemiri, Jean-Paul Dietz, Jack Sobel, Mondher Toumi

**Affiliations:** 1 Université Lyon I, decision sciences and health policies, Lyon, France; 2 Université EPHE, Paris, France; 3 Pevion Biotech AG, Ittigen/Bern, Switzerland; 4 Creativ-Ceutical SA, Paris, France; 5 HMAccess, Health and Market Access Strategies S.A.S, Menthon, France; 6 Division of Infectious Diseases, Department of Internal Medicine, Wayne State University, Detroit, USA

## Abstract

**Background:**

Recurrent vulvovaginal candidosis (RVVC) is a chronic condition causing discomfort and pain. Health status and health-related quality of life (HRQoL) in RVVC were never previously described using validated questionnaires. The objective of this study is to describe subjective health status and HRQoL and estimate health state utilities among women with RVVC.

**Methods:**

A cross-sectional online survey was conducted among women who reported having suffered four or more yeast infections over the past 12 months, in five European countries (France, Germany, Italy, Spain and the UK) and the USA. Index scores were derived from the EQ-5D, a questionnaire providing a single index value for health status. The SF-36 questionnaire was used for HRQoL assessment. Information on disease severity, treatment patterns and productivity was also collected.

**Results:**

12,834 members of online research panels were contacted. Among them, 620 women with RVVC (5%) were selected to complete the full questionnaire. The mean EQ-5D index score was 0.70 (95% confidence interval: [0.67, 0.72]) and the difference between women with a yeast infection at the time of questionnaire completion and other respondents was 0.05 (p = 0.47). The EQ-5D index score increased significantly with the time since last infection (p < 0.001). 68% of women reported depression/anxiety problems during acute episode, and 54% outside episodes, compared to less than 20% in general population (p < 0.001). All SF-36 domain scores were significantly below general population norms. Mental health domains were the most affected. The impact on productivity was estimated at 33 lost work hours per year on average, corresponding to estimated costs between €266/year and €1,130/year depending on the country.

**Conclusions:**

Subjective health status and HRQoL during and in between acute inflammatory episodes in women with RVVC are significantly worse than in the general population, despite the use of antifungal therapy. The average index score in women with RVVC is comparable to other diseases such as asthma or COPD and worse than diseases such as headache/migraine according to US and UK catalogs of index scores. The survey also revealed a significant loss of productivity associated with RVVC.

## Background

Recurrent vulvovaginal candidosis (RVVC) is a debilitating chronic infectious condition. It is defined as four or more acute inflammatory episodes of VulvoVaginal Candidosis (VVC), also known as vaginal yeast infection, within a year [1,2]. The prevalence of RVVC is estimated at 6-8% in women aged between 18 and 65 years in the USA and Western Europe, according to two large studies [3,4] and similar estimates can be derived from assessments of RVVC prevalence as proportion of VVC cases and studies on population-based VVC prevalence [3-8].

The main symptoms of yeast infections are inflammation, itching, an abnormal vaginal discharge and painful sexual intercourse and urination. Such symptoms cause variable but often severe discomfort and pain. Acute inflammatory episodes usually are treated with anti-fungal drugs of the azole class. They are efficient in clearing the acute infection, but are unable to prevent recurrences, which occur on average after a few months only. Guidelines from a number of medical associations recommend a long-term suppressive treatment regimen with an anti-fungal drug, usually fluconazole, for at least 6 months, off label [9,10], which can prevent recurrences for the duration of the therapy, whereas recurrence rates of 60-70% within 6 months after treatment cessation were reported [11,12]. A modified weaning scheme over 12–18 months achieved a lower recurrence rate (36%) within 6 months after complete treatment cessation [13]. The cost of long-term treatment has been estimated at AUD 900 ($ 862) in Australia [14]. Many RVVC patients turn to alternative remedies like yoghurt and vinegar which only have very short-term palliative effects [15,16].

Clinical impression is that RVVC patients, despite current treatment options, suffer from a substantially impaired health-related quality of life (HRQoL), but quantifiable evidence is scarce. *Nyirjesy et al.* applied several validated pain, stress and depression measurements to a population of physician-diagnosed patients (N = 38) and observed a proportion of 29% with a clinical depression [17]. *Mendling et al.* reported SF-36 scores and further HRQoL-related information from a longitudinal study on RVVC patients receiving different therapeutic treatments (N = 3x30), indicating that mental health was more affected than physical health [18].

To our knowledge, no other study has been conducted to elicit subjective health status or to assess global HRQoL of RVVC patients. In times of limited health care budgets, the availability of quantitative data on the medical need of a debilitating but not life-threatening condition, comparable across countries and indications, are a prerequisite for stakeholders and decision makers to engage into research and development of innovative treatment approaches.

The primary objective of this study was to assess subjective health status and HRQoL, and more specifically to estimate health state utilities, among women with RVVC, compared to general female population. Secondary objectives were to describe the impact of the disease on subjective health status during and in between acute inflammatory episodes, and to assess productivity and activity impairment.

## Methods

### Respondent recruitment and selection

A cross-sectional online survey was conducted in five European countries (France, Germany, Italy, Spain and UK) and USA using pre-existing market research panels. A target sample size of 100 respondents with RVVC was set for each country. Respondents were recruited using the Research Now family of panels which included 6,000,000 panellists [19]. The panellists received an incentive from €0.5 to €5 for their participation in the survey.

The questionnaire was initially developed and launched in the UK. It was first tested on 40 persons from the UK panel (soft launch). The purpose of this pilot phase was to check whether there was any obvious misunderstanding of questions or unexpected answers, and that the routing between questions was correct. Based on findings from the pilot study, minor corrections were made (described below), and other panel members from the UK were invited to participate in the survey until 100 full questionnaires were completed (full launch). After reviewing the collected data, the questionnaire was translated and launched in other countries. Any respondents with a time of completion lower than 33% of the RVVC sample median completion time were removed.

The questionnaire started with five screening questions to identify women with RVVC aged between 18 and 65 years who had been told at least once by a health care professional that they had a yeast infection, and who experienced at least four yeast infections over the past 12 months. Age quotas were used to ensure that the sample was representative of women with RVVC. The screening questions were slightly re-worded after the soft launch in the UK. It was initially asked UK participants if they had more than four episodes in the last 12 months, and the exact number of episodes was established later in the full questionnaire. Nine women had answered during the screening phase that they had more than four yeast infections in the last 12 months, but subsequently gave a number of infections lower than four in the main part of the questionnaire. We did not consider those women in our analysis and revised the questionnaire accordingly. In the final version, women were asked to state the exact number of episodes in the screening section, to determine their eligibility for completing the main questionnaire. The women who were identified with RVVC in the screening completed the main questionnaire immediately after answering the screening questions.

The main questionnaire started with two questions to determine whether the participant had an acute VVC episode at the time of answering the questionnaire, and if not, the time elapsed since the last episode.

### Health status and HRQoL measurement

As the primary objective was to compare subjective health status and HRQoL in women with RVVC to general population norms, generic instruments were used: the EQ-5D to elicit health state utilities [20] and the SF-36 to provide more detailed description of HRQoL [21,22]. EQ-5D is one of the most widely used preference-based instruments for the assessment of subjective health status enabling comparisons with other diseases, and helping payers to arbitrate on allocation of resources between different treatments, potentially in different therapeutic areas. This instrument is recommended by the UK National Institute for Health and Clinical Excellence (NICE) for eliciting utilities, i.e. weights used to estimate quality-adjusted life-years in the context of cost-effectiveness analysis [23]. EQ-5D is designed for self-completion by respondents and is suitable for use in postal or online surveys [24].

**Figure 1:**
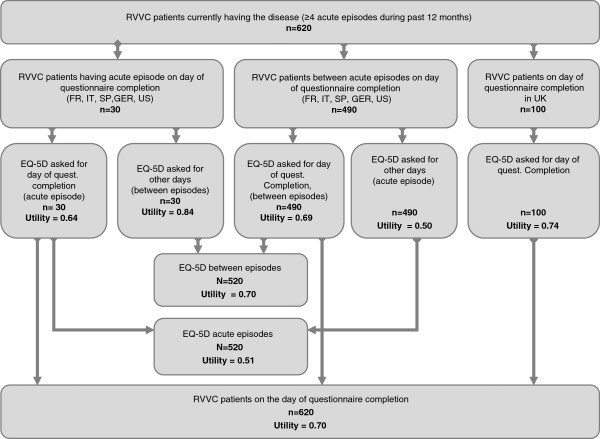
Mean EQ-5D utilities (based on UK TTO tariff) by patient subgroup, according to presence of symptoms on the day of questionnaire completion.

As it takes only a short time to complete, it was possible to ask the respondents to answer the EQ-5D twice. Firstly, respondents described their health state on the day of completing the questionnaire. Secondly, respondents who indicated having an acute episode on that day were asked to describe their health state on days without infection; others were asked to describe their health state on days with infection (Figure 1). Therefore, we were able to compare the health states during acute episode and between episodes for each woman in the last 12 months. However, these data were not used for the UK, because the question about having an episode at the time of completing the questionnaire appeared to be misunderstood. This question was rephrased before launching the study for other countries.

The SF-36 (version two) [21], another generic questionnaire, is more detailed than EQ-5D and frequently used to describe the disease’s burden in terms of HRQoL compared to the general population. It consists of 36 items, from which eight domain scores and two summary measures of health (the Physical Health Component Summary, PCS, and the Mental Component Summary, MCS) can be derived [25]. Validated translations are available for both EQ-5D and SF-36 for all the countries included in the study.

### Disease characteristics and management

To investigate the relationship between disease characteristics and HRQoL, another part of the questionnaire aimed to gather information on disease characteristics (frequency and duration of episodes, duration of disease) and disease management (history of long-term antifungal therapy, history of referral to a specialist).

### Work productivity and activity impairment

Questions on the impact on work and activities were directly adapted from a validated questionnaire (WPAI) [26] replacing “During the past seven days …” by “Thinking back to your most recent yeast infection…” and “…your health problems…” by “…your last infection…”. In the absence of validation of the questionnaire in French, German or Italian, the questions have been translated by native speakers and validated by experts.

The cost associated with work hours lost due to RVVC was estimated by country, by multiplying the corresponding number of lost work hours by the average hourly wage. Average national hourly wages were obtained using average annual wages in 2011 and average annual hours actually worked per worker [27] provided by the Organisation for Economic Co-operation and Development (OECD).

### Utility valuation

Utility scores were estimated during acute episodes and between acute episodes using EQ-5D, and were compared to general population scores from the literature.

Different value sets were available for EQ-5D, based on preference weights elicited in different countries [20]. In order to facilitate comparisons and generate an average score across all countries, preference weights elicited from the general population of the UK were first applied for all countries. The UK value set is the most widely used, and recommended by the EuroQol Group for use in cost-effectiveness/utility studies and comparative studies and to facilitate international comparisons [20,28]. These weights were generated from a large sample of the general population in the UK using the time-trade off (TTO) technique [29]. Utilities were also estimated based on preference weights elicited locally, in France, Germany, Spain and the USA. This was not possible for Italy where no set of values was developed.

### Statistical analysis

EQ-5D norms for proportions of patients reporting problems on each of the five EQ5D dimensions were reported by the EuroQol group [30] for the USA, Spain and Germany and by *König et al.*[29] for France and Italy. EQ-5D utility population norms were available for the US [31] and the UK [32,33], and calculated using proportions of patients by level on each dimension and country specific valuation formulas for other countries*.*

Utility values in general population were adjusted on the age distribution of the RVVC sample for Spain, Germany, US and UK but no age adjustments were made for France and Italy, as no breakdown by age group was found. However, *König et al*. analysed the effect of country on the occurrence of problems using multiple logistic regressions and controlling for socio-demographic variables. The paper presented odds ratio for different age categories. In a sensitivity analysis, age-adjusted population norms were obtained by applying these odds ratios on all dimensions of the EQ-5D.

Proportions of women reporting problems on each EQ-5D dimension were compared with population norms using the chi-square test of independence or Fisher’s exact test. Linear regression analyses on the EQ-5D utility at completion of the questionnaire were performed to determine the drivers of EQ-5D utility among disease-related variables, adjusting on patient characteristics.

For SF-36, individual scores can be compared with the population mean by transforming the individual score into standardized scores (Z-score and T-score) for each domain and summary component. As SF-36 norms were not found for most countries, the US population means and standard deviations were systematically used in the calculation of the standardized scores. After transformation, the average score in the general population was 50 and the standard deviation is 10. Whenever a domain or summary score was below 50, this indicated that HRQoL was worse in the studied population compared to the general population, and each point was one-tenth of a standard deviation. The correlation between EQ-5D dimensions and the SF-36 scales were calculated using the Spearman correlation coefficients.

## Results

### Patients & disease characteristics

12,834 members of online research panels were contacted and entered the self- screening questions. 865 women did not complete it and 4,389 women were not included in the study because the target number of respondents in their age group had been reached, not because they did not fulfil the criteria for RVVC. Of 7,580 (59%) women who completed the screening questions (excluding women out of the quotas), 639 (8.4%) were selected to complete the full questionnaire (5% of total contacted). The full questionnaire was completed by 620 women. Their mean age was 32 years (Table 1).

**Table 1:** Patient characteristics

		**UK**	**France**	**Spain**	**Italy**	**Germany**	**USA**	**Total**
	**N**	**100**	**107**	**100**	**105**	**106**	**102**	**620**
**Age**	Mean	30.3 (9.6)	32.3 (10.8)	32.1 (10.6)	32.1 (10.6)	31.4 (10.8)	32.1(10.8)	31.8 (10.5)
	Median (range)	27.0 [18.0; 56.0]	29.0 [18.0; 61.0]	29.0 [18.0; 60.0]	28.0 [19.0; 60.0]	28.0 [18.0; 62.0]	29.0 [18.0;63.0]	28.0 [18.0; 63.0]
**Marital status**	Single	37 (37.0%)	23 (21.50%)	23 (21.7%)	48 (45.7%)	37 (35.6%)	30 (29.4%)	195 (31.5%)
	Married	22 (22.0%)	33 (30.84%)	35 (33.0%)	32 (30.5%)	32 (30.8%)	50 (49.0%)	204 (32.9%)
	Divorced	2 (2.0%)	7 (6.54%)	3 (2.8%)	5 (4.8%)	1 (1.0%)	6 (5.9%)	23 (3.7%)
	Separated	1 (1.0%)	0	2 (1.9%)	0	4 (3.9%)	3 (2.9%)	10 (1.6%)
	Living with partner	38 (38.0%)	44 (41.1%)	43 (40.6%)	20 (19.1%)	30 (28.9%)	13 (12.8%)	188 (30.3%)
**Number of employed women**	N (%)	71 (71.0%)	83 (77.5%)	62 (62.0%)	60 (57.1%)	78 (73.5%)	69 (67.7%)	423 (68.2%)

Thirty women (5.8%) reported suffering a yeast infection on the day of completing the questionnaire, across all countries (except UK) and 20.6% had an infection less than a week ago. The mean duration of an acute episode was relatively homogeneous between countries, varying from 6.5 days to 7.4 days, apart from Spain (10.8 days). The average number of infections in the last 12 months was between five and six (Table 2). Overall, the periods with infection added up to 5.8 weeks on average over 12 months.

**Table 2:** Disease characteristics & treatment

		UK*	**France**	**Spain**	**Italy**	**Germany**	**USA**	**Total**
**Last time the women experienced the symptoms**	N		107	100	105	106	102	520
Today	N (%)		5 (4.7%)	4 (4.0%)	7 (6.7%)	7 (6.6%)	7 (6.9%)	30 (5.8%)
Less than a week ago	N (%)		27 (25.2%)	18 (18.0%)	20 (19.1%)	20 (18.9%)	22 (21.6%)	107 (20.6%)
1 to 4 weeks ago	N (%)		42 (39.3%)	49 (49.0%)	55 (52.4%)	42 (39.6%)	47 (46.1%)	235 (45.2%)
1 to 3 months ago	N (%)		24 (22.4%)	24 (24.0%)	15 (14.3%)	23 (21.7%)	24 (23.5%)	110 (21.2%)
3 to 6 months ago	N (%)		6 (5.6%)	4 (4.0%)	5 (4.8%)	10 (9.4%)	2 (2.0%)	27 (5.2%)
More than 6 months ago	N (%)		3 (2.8%)	1 (1.0%)	3 (2.9%)	4 (3.8%)	0	11 (2.1%)
**Symptoms today: n (%)**	N		5	4	7	7	7	30
Vaginal itching and/or Burning	N (%)		5 (100.0%)	4 (100.0%)	7 (100.0%)	6 (85.7%)	4 (57.1%)	26 (86.7%)
Thick, white or yellowish vaginal discharge	N (%)		4 (80.0%)	4 (100.0%)	6 (85.7%)	4 (57.1%)	5 (71.4%)	23 (76.7%)
Pain on intercourse	N (%)		2 (40.0%)	3 (75.0%)	2 (28.6%)	1 (14.3%)	3 (42.9%)	11 (36.7%)
Pain and burning on urination	N (%)		0	1 (25.0%)	4 (57.1%)	3 (42.9%)	4 (57.1%)	12 (40.0%)
**Number of infections**								
	N	100	107	100	105	106	102	620
	Mean (SD)	5.74 (2.19)	5.22 (1.43)	5.22 (1.45)	5.28 (1.54)	5.05 (1.26)	5.60 (1.81)	5.34 (1.64)
**Duration of infection (days)**								
	N	100	107	100	105	106	102	620
	Mean (SD)	6.96 (5.00)	7.01 (4.31)	10.75 (12.25)	7.43 (4.94)	6.50 (4.76)	6.66 (4.65)	7.57 (6.77)
**Patients treatment with long-term antifungal therapy**	N	100	107	100	105	106	102	620
Patients never treated	N (%)	68 (68.00%)	45 (42.06%)	45 (42.45%)	29 (27.62%)	72 (69.23%)	64 (62.75%)	321 (51.8%)
Patients currently treated	N (%)	12 (12.00%)	29 (27.10%)	25 (23.81%)	42 (40.38%)	9 (8.49%)	26 (25.49%)	141 (22.7%)
Previously treated	N (%)	20 (20.00%)	33 (30.84%)	36 (33.96%)	34 (32.38%)	23 (22.11%)	12 (15.79%)	158 (25.5%)

*Data about symptoms not available for the UK, as questions about symptoms were formulated differently in the UK version.

48% of the RVVC women (N = 299) had been treated with a long-term antifungal treatment at least once (Table 2). Among them, 141 were treated at the time they answered the questionnaire, and 158 were treated previously. Among the 158 women previously treated with long-term antifungal, 121 (77%) completed the normal course of the treatment and 92 (61%) had at least one relapse since stopping long-term antifungal treatment. 75% of the women reported they had seen a gynaecologist.

### Impact of RVVC on subjective health status and HRQoL

The proportion of women with problems related to pain/discomfort of EQ-5D was 63% on average (from 55% in Germany to 78% in Italy) whereas the proportions in the general population did not exceed 35% (from 23% in Spain to 35% in France). Anxiety/depression was also significantly affected, with 53% of women reporting some or severe problems on that dimension (from 43% in UK to 62% in Italy), compared to below 20% in the general population. Substantial differences between our sample and the general population were also found for problems related to usual activities. Differences vs. the general population were significant for all dimensions (see Table 3).

**Table 3:** Proportions by EQ-5D dimensions and correlation with SF-36

		**Number of women**	**EQ-5D mobility**	**EQ-5D self-care**	**EQ-5D usual activities**	**EQ-5D pain/discomfort**	**EQ-5D anxiety/depression**	**Source**
**Proportions of patients with problems at the completion day**								
**UK**	RVVC sample	N = 100	17.0%	8.0%	24.0%	60.0%	43.0%	
	General population	N = 1926	13.4%	3.1%	11.9%	25.4%	18.4%	*euroqol*
	Test (p-value)		P < 0.0001	P < 0.0001	P < 0.0001	P < 0.0001	P < 0.0001	
**France**	RVVC sample	N = 107	12.2%	5.6%	17.8%	65.4%	57.0%	
	General population	N = 2,892	13.2%	4.1%	10.0%	35.3%	14.6%	*Konig et al.*
	General pop (adjusted)	N = 2,892	17.1%	5.4%	13.5%	49.0%	21.8%	
	Test (p-value)		P < 0.0001	P < 0.0001	P < 0.0001	P < 0.0001	P < 0.0001	
**Spain**	RVVC sample	N = 100	20.0%	20.0%	29.0%	60.0%	49.0%	
	General population	N = 323	5.9%	0.4%	5.5%	22.9%	18.1%	*euroqol*
	Test (p-value)		P < 0.0001	P < 0.0001	P < 0.0001	P < 0.0001	P < 0.0001	
**Italy**	RVVC sample	N = 105	35.2%	22.9%	56.2%	78.1%	61.9%	
	General population	N = 4,709	12.0%	4.3%	10.9%	27.6%	9.3%	*Konig et al.*
	General pop (adjusted)	N = 4,709	15.6%	5.7%	14.7%	38.3%	13.9%	
	Test (p-value)		P < 0.0001	P < 0.0001	P < 0.0001	P < 0.0001	P < 0.0001	
**Germany**	RVVC sample	N = 106	17.0%	5.7%	25.5%	54.7%	50.9%	
	General population	N = 340	7.4%	1.1%	6.7%	27.4%	19.6%	*euroqol*
	Test (p-value)		P < 0.0001	P < 0.0001	P < 0.0001	P < 0.0001	P < 0.0001	
**USA**	RVVC sample	N = 102	29.4%	20.6%	33.3%	57.8%	55.9%	
**Total**	RVVC sample	N = 620	17.0%	5.7%	25.5%	54.7%	50.9%	
**Spearman correlation coefficients with:**								
Physical health - SF36	Correlation coeff.		**−0.41027**	**−0.37495**	**−0.41759**	**−0.39627**	−0.21067	
	P-value		<.0001	<.0001	<.0001	<.0001	<.0001	
Mental health - SF36	Correlation coeff.		−0.2122	−0.18161	−0.20143	−0.22917	**−0.44891**	
	P-value		<.0001	0.0002	<.0001	<.0001	<.0001	

The average utility score for women with RVVC across six countries was estimated at 0.70 on the day of completion of the questionnaire (Table 4). The utilities obtained using the country-specific preference weights were significantly lower than the age-adjusted general population, with a difference vs. general population norms ranging from 0.08 in Germany to 0.21 in Spain (Table 4).

**Table 4:** EQ-5D utilities

		**UK**	**France**	**Spain**	**Italy**	**Germany**	**USA**	**Total**
**All RVVC sample, during the day of completion of the questionnaire (a)**								
	N	100	107	100	105	106	102	620
(UK TTO formula)	Mean (Sd)	0.74 (0.28)	0.72 (0.25)	0.67 (0.34)	0.61 (0.30)	0.76 (0.25)	0.68 (0.34)	0.70 (0.30)
(country specific formula)	Mean (Sd)	0.74 (0.28)	0.77 (0.23)	0.72 (0.32)	-	0.86 (0.20)	0.76 (0.23)	-
**Women with infection, during the day of completion of the questionnaire**								
	N		5	4	7	7	7	30
(UK TTO formula)	Mean (Sd)	-	0.62 (0.41)	0.48 (0.30)	0.73 (0.10)	0.79 (0.34)	0.53 (0.33)	0.64 (0.31)
(country specific formula)	Mean (Sd)	-	0.78 (0.22)	0.73 (0.33)	-	0.87 (0.20)	0.77 (0.23)	-
**Women Without infection, during the day of completion of the questionnaire**								
	N		102	96	98	99	95	490
(UK TTO formula)			0.73 (0.25)	0.68 (0.34)	0.60 (0.31)	0.75 (0.24)	0.69 (0.34)	0.69 (0.30)
(country specific formula)	Mean (Sd)		0.78 (0.22)	0.73 (0.33)	-	0.87 (0.20)	0.77 (0.23)	-
**General population (b)**								
	N	3395	2892	5473	4709	3552	4000	-
	Mean (Sd)	0.92 (0.22)	0.89 (−)	0.93 (−)	0.90 (−)	0.94 (−)	0.88 (−)	0.91 (−)
	Adjusted mean (Sd)	-	0.86 (−)	0.89 (−)	0.87 (−)	0.92 (−)	-	-
	Source	*Dennis et al.*	*König et al.*	*König et al.*	*König et al.*	*König et al.*	*Kind et al.*	-
**Difference (a - b)**								
(UK TTO formula)	Mean (Sd)	−0.18 (−)	−0.17 (−)	−0.26 (−)	−0.29 (−)	−0.18 (−)	−0.20 (−)	−0.21 (−)
	Adjusted mean (Sd)	-	−0.14 (−)	−0.22 (−)	−0.26 (−)	−0.16 (−)	-	-
(country specific formula)	Mean (Sd)	−0.18 (−)	−0.12 (−)	−0.21 (−)	-	−0.08 (−)	−0.12 (−)	-
	Mean (Sd) (adjusted)	-	−0.09 (−)	−0.17 (−)	-	−0.06 (−)	-	-
During the last acute episode (at the completion of the questionnaire or before) (c)*								
	N	100	107	100	105	106	102	520
(UK TTO formula)	Mean (Sd)	0.71 (0.27)	0.52 (0.33)	0.48 (0.34)	0.42 (0.35)	0.62 (0.28)	0.51 (0.36)	0.51 (0.34)
(country specific formula)	Mean (Sd)	0.71 (0.27)	0.59 (0.31)	0.54 (0.35)	-	0.76 (0.24)	0.64 (0.24)	-
Symptom-free period (at the completion of the questionnaire or before) (d)*								
	N	100	107	100	105	106	102	520
(UK TTO formula)	Mean (Sd)	0.86 (0.24)	0.72 (0.26)	0.68 (0.34)	0.62 (0.31)	0.77 (0.24)	0.69 (0.33)	0.70 (0.30)
(country specific formula)	Mean (Sd)	0.86 (0.24)	0.77 (0.23)	0.73 (0.32)	-	0.87 (0.19)	0.77 (0.23)	-
Difference (c - d)*								
	N	100	107	100	105	106	102	520
Utility score (UK TTO formula)	Mean (Sd)	−0.15 (0.23)	−0.20 (0.29)	−0.20 (0.34)	−0.20 (0.30)	−0.15 (0.23)	−0.18 (0.27)	−0.19 (0.29)
Utility score (country specific formula)	Mean (Sd)	−0.15 (0.23)	−0.19 (0.25)	−0.19 (0.32)	-	−0.12 (0.20)	−0.12 (0.18)	-

*Total do not include data from the UK, as questions about symptoms were formulated differently in the UK version.

**Figure 2:**
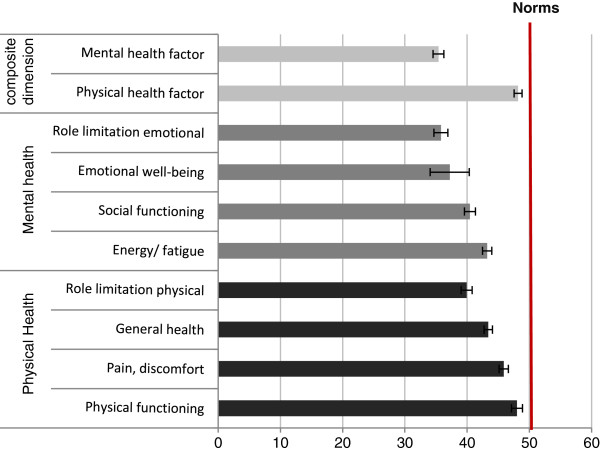
**SF-36 T-scores for each dimension.** All scales in the general population have the same average (50) and the same standard deviation (10).

All SF-36 domain scores, presented in the Figure 2, were significantly lower among women with RVVC than in the general population. Results were homogeneous across countries with T-scores varying from 34 to 37 for mental health scores and from 45 to 46 for the pain scores. The most significantly affected domains were those related to mental health, particularly the “emotional well-being” and “role limitation emotional” domains. The average mental component summary score was estimated at 34.72 (95% CI: [33.74; 35.71]).

### Health state utility values during and outside infection periods

Analyses comparing health state utilities during and outside infection periods were conducted for all countries except the UK (Figure 1 and Table 4). The average utility during the last infection, whenever it occurred (during the completion of questionnaire or before), was lower than the utility outside infection (0.51 vs. 0.70; Table 4). 90.8% of women had pain/discomfort problems during the last episode, vs. 60.8% outside infections, (p < 0.0001), on average across five countries. Differences were smaller on the anxiety/depression dimension, as many women reported suffering anxiety or depression between acute episodes.

The difference was smaller when separating women with or without infection at the time of completing the questionnaire: the utility score was estimated at 0.69 without infection vs. 0.64 with infection (p < 0.001).

The regression model in Table 5 shows that respondents with a longer time since last symptoms had a higher utility on the day of questionnaire completion. For example, the difference in utility between women without symptoms for three months and those with symptoms in the past week was 0.1633 (p = 0.0033), all other things equal. In addition, women undergoing long-term antifungal therapy had a significantly lower utility compared to those treated in the past or never treated. The effect of the time since the disease onset was tested but this variable was not included in the final model as it did not impact significantly the EQ-5D utility.

**Table 5:** Multivariate regression on the EQ-5D utility today (except UK)

	**Category**	**Reference**	**Estimates**	**P-value**
**Age (years)**			−0.0021	0.0779
**The last time you experienced symptoms**	1 to 4 weeks ago	This week	0.1161	0.0004
	1 to 3 months		0.1507	0.0001
	More than 3 months		0.1633	0.0033
**Number of yeast infection**	4-5 infections	6 infections and more	0.0507	0.0762
**When did you stop the antifungal long-term therapy?**	In the past 12 months	This treatment is still ongoing	0.1566	0.0002
	More than 12 months ago		0.2249	0.0001
	Never treated		0.2081	<.0001

**Figure 3:**
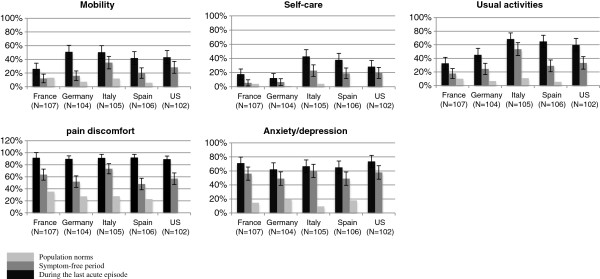
Proportion of women with some/severe problems during the last acute infection/outside infection.

The health status of women with RVVC was not only lower than the general population during an acute episode, but also outside infections. Large differences in the proportions of subjects reporting problems were found for the anxiety/depression dimension, between women with RVVC during infection-free periods (54%) and women in general (≤20%). The difference was largest in Italy, where 60% reported anxiety/depression problems outside episodes vs. 9.3% in the general population (Figure 3).

### Work productivity and activity impairment

Around 50% of the RVVC sample claimed the disease impacted their daily normal activity (from 45% for UK to 60% for USA). In addition, working women with RVVC missed around six hours of work per episode of yeast infection (Table 6). Therefore, the individual number of missing hours (hours per episode * episodes per year) was estimated at around 33 hours per year. The costs of lost productivity over 12 months were estimated at €1,130/per year for France, €852/per year for the UK (i.e. £713) and €974/per year for US (i.e. $1,261).

**Table 6:** Productivity lost

	**UK**	**France**	**Spain**	**Italy**	**Germany**	**USA**	**Source**
	100	107	100	105	106	102	
Number of employed women	71 (71.0%)	83 (77.5%)	62 (62.0%)	60 (57.1%)	78 (73.5%)	69 (67.7%)	
Missing hours from work/RVVC episode	5.86 (12.39)	8.72 (17.67)	3.37 (9.62)	5.23 (8.29)	6.12 (10.59)	7.00 (10.80)	
Number of missing hours/year	33.6	45.5	17.6	27.6	30.9	39.2	
Average annual hours actually worked per worker - 2011 (b)	1625	1476	1690	1774	1413	1787	OECD
Average annual wages (National Currency Unit) - 2011 (c)	31413	34284	27010	28109	33766	54450	OECD
Average daily wages (National Currency Unit) - 2011 (d) = (c)/(b)	19.33	23.23	15.98	15.84	23.9	30.47	
Average daily wages. in €	19.33*	23.23	15.98	15.84	23.9	23.53**	
**Monetary equivalence of the annual productivity loss (€) (mean.sd)**	**851.6 (1580)**	**1129.9 (2316)**	**265.7 (677)**	**455.1 (679)**	**785.0 (1384)**	**973.9 (1613)**	

*Conversion rate: 31/12/2011: 1.193.

**Conversion rate: 31/12/2011: 0.7722.

### Correlations between instruments

Correlations between SF-36 summary scales and EQ-5D ratings by dimension were weak to moderate. The SF-36 mental component summarily was moderately correlated with the EQ-5D anxiety/depression dimension (Spearman’s correlation: 0.45), and weakly correlated with other dimensions (0.18 to 0.23). On the opposite, the SF-36 physical component summary was moderately correlated with mobility, self-care, usual activities and pain/discomfort (0.37 to 0.42), and weakly with anxiety/depression (0.21).

The number of missing hours was strongly associated with the EQ-5D, specifically with ratings of problems with usual activities.

## Discussion

The main objective of the survey was to elicit utility values for RVVC. The EQ-5D questionnaire, a widely used and recommended generic questionnaire for utility value elicitation, was used to quantify the impact of RVVC. This also allowed for comparison with general population norms and other diseases. The SF-36, another generic questionnaire, was used to describe in more detail the impact of RVVC in HRQoL.

The average health state utility for women with RVVC was estimated at 0.70 (based on the UK TTO formula), despite the use of long term antifungal therapy in 85% of participants. According to US and UK catalogues of utilities, this situates RVVC as worse than headache/migraine for example, and similar to asthma or COPD [34,35]. The difference *vs*. age-adjusted population norms was estimated at −0.21 globally, based on the UK tariff, and ranged from −0.12 for France to −0.21 for Spain, using tariffs reflecting local preference weights. A minimal clinically important difference of 0.074 has been reported in the literature [36]. Thus, the utility decrement among women with RVVC was found to be clinically important for all countries.

SF-36 dimensions were all affected among women with RVVC in general. The greatest impact of the disease was found on the emotional domains compared to the general population, but significant differences were found for other domains as well. The summary scores of the SF-36 also suggested that mental health was strongly affected by RVVC (mean score: 34.72; 95% CI: [33.74; 35.71] for all countries), which was consistent with the results from EQ-5D. This outcome was consistent with findings by *Mendling et al.*, who also reported a stronger impact on mental health than on physical health using SF-36, albeit to a somewhat lesser extent [18].

Acute RVVC episodes impacted subjective health status negatively, but elicited health status was also affected outside RVVC episodes notably due to a large impact of the disease on anxiety or depression that lasts over time. Stress and a substantial psychological burden that is associated with the disease, up to depression, has been described previously and confirms the findings of the present study [17,37,38]. It is however not clear to what extent stress, anxiety and depression are causes or symptoms of RVVC, or both in an amplifying loop.

The impact of RVVC on subjective health status and HRQoL exists in spite of existing treatments, such as short- or long-term antifungal therapy. Many participants had previously received long-term antifungal therapy. Women who were receiving a long-term antifungal therapy at the time of the study had worse subjective health status compared to others; this was likely related to the fact that those who sought treatment were the women in whom subjective health status was most affected.

The impact on productivity, estimated at 33 work hours lost per year on average, was high. Associated costs ranged from €266/year for Spain to €1,130/year for France. These values were higher than expected, but they seemed credible as there was good internal consistency between the rating of usual activities and the number of missed work hours per episode from WPAI.

The survey was conducted in a large sample of women (n = 620), covering six countries. Despite variability in health state utility values and HRQoL scores between countries, the key findings were consistent between countries. Differences in utility values and HRQoL scores between RVVC and the general population were always significant and clinically important. In addition, the correlations between EQ-5D and SF-36 were in line with expectations: the EQ-5D physical dimensions were mostly correlated to SF-36 physical domains and the EQ-5D anxiety/disorder rating was mostly correlated to SF-36 mental domains. The extent of correlation between EQ-5D and SF-36 was consistent with previous studies, also reporting low to moderate correlations between the two instruments (for example in low back pain [39]).

A limitation of this study was that data on co-morbidities was not available. It is possible that the difference in utility between women with RVVC and the general population was partially attributable to co-morbidities with higher occurrence in RVVC patients than in the general population [1,2]. In some rare cases, yeast infection may be an early sign of diabetes [40]. This could explain why there were more women with RVVC reporting problems on dimensions such as mobility, outside infection periods. However, regression analysis demonstrated that utility worsens with increasing disease severity, and therefore, that the estimated reduction in utility was at least partially attributable to RVVC itself. In addition, vestibular vulvodynia, is often present in RVVC patients. The causal relationship between RVVC and vestibular vulvodynia is not clear. Vestibular vulvodynia could be a symptom of RVVC or a comorbidity triggered by RVVC as suggested by a recent report [41], although this pain syndrome may also have other origins.

The study was also limited by the fact that online self-reported answers could not be verified, in particular the diagnosis. However, women were not asked to state directly, whether they had RVVC or not. The screening section was designed in a way that respondents did not know that the topic was RVVC until the completion of all five screening questions, where they were asked about a physician-diagnosed episode of VVC (or suggested synonyms) and the number of such episodes over the past 12 months. Although women could receive a small financial incentive to participate, (from €0.5 to €5), this incentive was sufficiently small not to attract “false respondents”, who would participate only in order to receive the financial incentive.

The proportion of women with symptoms on the day of the questionnaire (5.8%) was lower than expected (according to average number of days and average duration of an episode). Potential reasons for this relatively low proportion may be that symptoms are not continuous during acute episodes, or that women with RVVC are less likely to participate in online surveys during acute episodes. However, the sum of the number of women with an acute episode at the time of questionnaire completion or in the past week was consistent with the number of episodes per year and the duration of an acute episode.

A minor limitation for the UK sample was that the question about the presence of symptoms on the day of completing the questionnaire appeared to be misunderstood in the soft launch and therefore had to be rephrased before launching the surveys in other countries. Utilities for acute episodes and periods in between episodes could thus not be separated for the UK. However all other parts of the questionnaire were identical, and global RVVC utilities on the day of questionnaire completion are comparable between the UK and other countries.

When testing for the significance between utilities among women with RVVC and the general population, the standard error around utility norms was ignored. Standard errors around the utility norms were not available for some countries, but these were known to be small compared to estimated differences between women with RVVC and women in general for the UK and the US. In the UK, the standard error around the utility norm for women were 0.005 (SD = 0.22; N = 1,925). In the US, standard errors of scores in each age or gender category were around 0.02.

Another limitation was the calculation of the utility during acute episodes by pooling women having an acute episode at completion of the questionnaire with women recalling the last acute episode (having occurred on average 2.2 months ago, but in any case within the past 12 months). It was uncertain how well women could recall the quality of life during the last episode. We noted, however, that the reported difference in EQ-5D utility during and between episodes was very similar for patients who answered the questionnaire during an acute episode and those who answered in the interval between episodes (0.20 and 0.19, respectively).

We used modified versions of the WPAI questionnaire. Questions were reworded replacing “During the past seven days …” by “Thinking back to your most recent yeast infection…” and the “…your health problems…” by “…your last infection…”. The WPAI questionnaire, while being validated in English and Spanish, has not been validated in German, French or Italian placing a potential limitation on the accuracy of the data gathered from respondents from these countries. However, all translations were carefully reviewed by native speakers and clinicians.

## Conclusions

This survey showed that subjective health status and HRQoL are substantially diminished during acute episodes, but also outside these episodes in women with RVVC, including some women who have received long-term antifungal therapy as recommended by guidelines. It appears that many women with RVVC suffered some discomfort, and most importantly anxiety between acute episodes. The survey also revealed that the disease has a strong impact on patient’s usual daily activities and work. RVVC was found to be associated with significant productivity costs. These findings highlight the need for more effective ways to manage RVVC.

## Endnotes

^a^The proportion of women reporting having symptoms while answering the questionnaire (80%) was much higher than expected in the UK. The question was probably misunderstood. Based on the UK results, the questions on symptoms were revised before the launch in other countries.

## Competing interests

The authors declare that they have no competing interests.

## Authors’ contributions

SA participated in the conception of the study and the questionnaire, supervised the analysis, contributed in the interpretation of data and revised the manuscript critically. FG participated in the design of the questionnaire, supervised the analysis, interpreted the data and drafted the manuscript. AK performed the data management and the statistical analysis and contributed in the interpretation of the data. JW participated in the conception of the study, revised the manuscript critically for important intellectual content and gave final approval of the version to be published. JPD participated in the conception of the study, revised the manuscript critically for important intellectual content and gave final approval of the version to be published. JS contributed in the interpretation of data and revised the manuscript critically for important intellectual content. MT revised the manuscript critically for important intellectual content and gave final approval of the version to be published. All authors read and approved the final manuscript*.*
